# Assessing Nordihydroguaiaretic Acid Therapeutic Effect for Glioblastoma Multiforme

**DOI:** 10.3390/s22072643

**Published:** 2022-03-30

**Authors:** Felicia S. Manciu, Jose Guerrero, Kevin E. Bennet, Su-Youne Chang, Masum Rahman, Lizbeth V. Martinez Lopez, Siobhan Chantigian, Mariana Castellanos, Marian Manciu

**Affiliations:** 1 Department of Physics, University of Texas at El Paso, El Paso, TX 79968, USA; jaguerrero9@miners.utep.edu (J.G.); lmartinezl@miners.utep.edu (L.V.M.L.); mcastellano@miners.utep.edu (M.C.); mmanciu@utep.edu (M.M.); 2 Border Biomedical Research Center, University of Texas at El Paso, El Paso, TX 79968, USA; 3 Department of Neurologic Surgery, Mayo Clinic, Rochester, MN 55905, USA; kbennet@mayo.edu (K.E.B.); Chang.SuYoune@mayo.edu (S.-Y.C.); Rahman.Masum@mayo.edu (M.R.); Chantigian.Siobhan@mayo.edu (S.C.); 4 Division of Engineering, Mayo Clinic, Rochester, MN 55905, USA; 5 Department of Physiology and Biomedical Engineering, Mayo Clinic, Rochester, MN 55905, USA

**Keywords:** Raman microscopy, optical, statistical analysis, principal component analysis, glioblastoma cells, nordihydroguaiaretic acid, tumor inhibition, drug assessment, healthcare sensing

## Abstract

In this study, we demonstrate that Raman microscopy combined with computational analysis is a useful approach to discriminating accurately between brain tumor bio-specimens and to identifying structural changes in glioblastoma (GBM) bio-signatures after nordihydroguaiaretic acid (NDGA) administration. NDGA phenolic lignan was selected as a potential therapeutic agent because of its reported beneficial effects in alleviating and inhibiting the formation of multi-organ malignant tumors. The current analysis of NDGA’s impact on GBM human cells demonstrates a reduction in the quantity of altered protein content and of reactive oxygen species (ROS)-damaged phenylalanine; results that correlate with the ROS scavenger and anti-oxidant properties of NDGA. A novel outcome presented here is the use of phenylalanine as a biomarker for differentiating between samples and assessing drug efficacy. Treatment with a low NDGA dose shows a decline in abnormal lipid-protein metabolism, which is inferred by the formation of lipid droplets and a decrease in altered protein content. A very high dose results in cell structural and membrane damage that favors transformed protein overexpression. The information gained through this work is of substantial value for understanding NDGA’s beneficial as well as detrimental bio-effects as a potential therapeutic drug for brain cancer.

## 1. Introduction

Although the death rate due to cancer has continuously declined in the United States since 1990 [1], this threat to human health is still significant. One known difficulty for effective cancer therapy is that the same tumor can exhibit different phenotypes when growing in different organs, randomly affecting cancer progression and hindering the prevention of metastases [2,3,4,5,6]. This challenge is further amplified by significant variability in the metastatic latency period, which can vary from months to years depending on the affected organ (e.g., breast, liver, lung, bone, and brain) [2,3,4,5,6]. Another significant obstacle to efficient cancer treatment is therapeutic resistance. It has been suggested that the most beneficial treatments should mediate multi-organ metastasis, not just target organ-specific malignant tumors [2,6].

Typically, 85% to 90% of brain tumors form in the central nervous system from neoplasm and metastatic cancer cells that travel from other organs through the blood [7]. Malignant gliomas, which include the intracranial glioblastoma multiforme (GBM) that is analyzed in this work, occur in more than 70% of the cases [8]. Of all the malignant gliomas, GBM is the most common and most lethal primary brain tumor, with a high rate of recurrence [9,10]. While the causes of this intracranial type of brain cancer remain largely unknown, due to its aggressiveness, patients diagnosed with GBM have a 10% prognosis of surviving for 5 years [11,12]. Current standard care consists of maximum surgical resection, followed by radiation and treatment with temozolomide [7,8,9]. The highly infiltrative nature of this tumor invariably leaves residual tumor cells that might become resistant to chemotherapy and make recurrence inevitable. It is also known that recurrent tumors can be more damaging and therapy-resistant than the original malignancy [13,14,15]. Despite all the recent advances in cancer therapeutics, the various pathways and mechanisms of cancer occurrence, resistance, and recurrence are challenges that remain to be overcome. Consequently, there is still an acute need for new tools and approaches that examine existing problems from new perspectives, and that provide insights into biochemical mechanisms at the cellular level.

Another important factor in improving the overall survival (OS) rate is reliable and early disease detection and diagnosis. The most commonly employed clinical diagnostic methods for brain tumors are magnetic resonance imaging (MRI), computed tomography (CT), positron emission tomography (PET), and ultrasound sonography (US). Typically, such imaging procedures are performed before and after the surgical tumor resection. Standard histopathology follows to achieve a biochemical and morphological assessment of the retrieved tissue. Accurate and rapid results can also be obtained using optical technologies [16,17,18], which have evolved since 1984 [19]. Their effectiveness as biomedical tools for in vitro and in vivo cancer assessment and in viewing cellular morphological transitions during complex biological processes has improved significantly over time [16,18,20,21,22,23,24]. Raman microscopy, an optical technique that not only has all the benefits of optical methods employed for real-time diagnosis but also has the important advantage of not requiring fluorescent tagging for detecting molecular signatures, has recently been garnering attention [18]. Particular to the advances in Raman technique is the achievement of brain cancer cell detection in humans with an accuracy that is beyond the capability of MRI [18]. Computational algorithms have also become important contributors to the overall progress in health diagnosis.

The current analysis, in addition to discriminating between malignant and benign biospecimens, which has already been accomplished using Raman spectroscopy [18,20,21,22,23,24], targets accurate identification of morphological changes occurring in GBM bio-signatures upon treatment with nordihydroguaiaretic acid (NDGA). From the many natural products and their chemical extracts traditionally used as remedies by the general public, we consider, in this study, the phenolic lignan NDGA, since it is the main chemical extract from a plant native to the region encompassing El Paso, Texas. Locally, the plant is known as *la Gobernadora* or *Creosote* bush. The Latin name of the plant is *Larrea tridentata*. Due to its widespread use for alleviating a variety of illnesses, which includes cancer, the plant has been extensively studied [25,26,27,28,29,30,31,32,33,34,35,36,37,38,39,40,41,42]. The extraction, synthesis, and purification of the plant’s most beneficial active ingredient, the NDGA [26,27], has opened new research opportunities, with abundant literature dedicated to scientifically ascertaining and clarifying the compound’s potential value and underlying mechanisms of action. Examples consist of preventing kidney stone formation, diabetes, renal infections, neurological disorders, viral infections, and a variety of cancer types [28,29,30,31,32,33,34,35,36,37,38,39,40,41,42]. It has been established that the NDGA’s antioxidant, anti-viral, and anti-tumorigenic properties are due to the fact that it is a reactive oxygen species (ROS) scavenger, leading to a decrease in inflammation and to the inhibition of several metabolic enzymes and receptor tyrosine kinases [28,29,30,31,32,33,34,35,36,37,38,39,40,41,42]. It has also been reported that excessive consumption of the plant or administration of the compound at high concentrations can be detrimental and toxic, causing liver damage and kidney dysfunction [29]. The cytotoxic effects of NDGA have been attributed to the molecule’s auto-oxidation and its structural transformation into semi-quinone or ortho-quinone forms (the NDGA molecule contains two catechol rings, which are prone to oxidation) [30,40]. Although the majority of these studies were preclinical ([30] and references therein), NDGA’s known rapid auto-oxidation and complex redox process precluded support for clinical translation of the compound. Furthermore, distinguishing the various structural configurations of NDGA by standard fluorescence microscopy is hampered by the fact that all of their UV emissions overlap closely at about 280 nm.

While NDGA administration in high doses of more than 100 mg/kg for prolonged periods of time is detrimental [30], it may have some beneficial chemotherapeutic effects when provided as bolus therapy (a high dose over a short time). The literature reports successful in vitro use of NDGA for the treatment of lung and breast cancer [30,37,41,42], which are contributors to malignant brain tumors and metastasis. Since NDGA could be a therapeutic compound that effectively mediates and inhibits the formation of multi-organ malignant tumors [30,37,41,42], in this study, we investigate its bio-effects on the intracranial GMB brain tumor. The analysis of in vitro bio-structural modifications of untreated and NDGA-treated GMB cells is performed through a combined experimental Raman microscopy and computational approach. The simultaneous changes experimentally observed for Raman vibrational signatures of proteins, lipids, and nucleic acids are accurately discriminated by statistical analysis. The information achieved, which can be correlated with NDGA’s bio-activity, is of substantial value for decoding the compound’s therapeutic mechanisms of action and developing and/or screening novel pharmacological applications in brain cancer treatment.

## 2. Materials and Methods

### 2.1. Sample Preparation

The human glioblastoma GBM6 cell line from the Mayo Clinic’s National patient-derived xenografts repository in Rochester, Minnesota, was used in this study. Short-term explant culture protocols were previously published [43]. The cells were further plated in culture flasks (250 mL, Falcon, Corning, NY, USA) for proliferation. Once the confluence of the cells reached 50%, the cells were washed with phosphate buffer (PBS, 4–5 mL, pH 7.0; Roche Life Science, Mannheim, Germany) and detached from the flask by incubation in a trypsin-EDTA solution (37 °C, 95% O_2_/5% CO_2_) for a couple of minutes. To neutralize the trypsin, after cell detachment, fetal bovine serum (FBS, 10%, Thermo Fisher Scientific Inc., Waltham, MA, USA) was also added. A standard culture medium of a mixture of high glucose Dulbecco’s Modified Eagle’s Medium (DMEM, 500 mL; Gibco, Waltham, MA, USA), FBS (10%, 50 mL; Gibco, Waltham, MA, USA), and penicillin-streptomycin (1%, 5 mL; Gibco, Waltham, MA, USA) was prepared and added into the flask. The mixture containing the isolated GBM cells was then transferred to a 50 mL centrifuge test tube and spun down at 1200 rpm for 10 min. The supernatant was removed and the cells were resuspended in 1.0 mL DMEM. The cell viability and their numbers were checked using the trypan blue staining method (0.4%). To clarify the media, the cells were resuspended with additional DMEM (17 mL) and 3 mL of the suspended cells were plated onto coverslips, which were autoclaved and coated with poly-L lysine (1:10, final concentration). After cell attachment to the coverslips, they were returned to the incubator (37 °C, 5% CO_2_). A MycoAlertTM Mycoplasma Detection Kit (catalog #: LT07-118; Lonza, Rockland, MI, USA) was used to test cell supernatants for mycoplasma contamination at regular intervals, and the results were negative. Short tandem repeat analysis was used to ensure cell authenticity in each experiment when compared with historical controls.

The following protocol was employed for the cell treatment with NDGA. First, 1M stock solution was made by adding 30 mg of NDGA (Sigma-Aldrich, St. Louis, MO, USA) into 90 μL dimethylsulfoxide solution (DMSO; Life Technologies, Carlsbad, CA, USA). Once the NDGA powder was fully dissolved, the final volume was adjusted to 1 mL. For Raman studies, this solution was further diluted in cell media to 100 µM and 250 µM NDGA concentrations and used immediately to avoid potential oxidation. The low-dosed GBM6 cells were incubated for 24 h in a 100 µM NDGA concentration and the high-dosed cells for 4 h in a 250 µM concentration. After the incubation, the cells were washed five times with PBS and fixed on plain microscope glass slides with a 4% paraformaldehyde solution (Beantown Chemical, Hudson, NH, USA) for 15 minutes. Then, the cells were washed five times with PBS followed by five washes with doubly distilled water and allowed to air dry at room temperature.

### 2.2. Instruments

The confocal Raman images of 55 µm × 55 µm scan sizes were acquired with an alpha 300RAS WITec system (WITec GmbH, Ulm, Germany), which consists of a microscope coupled via an optical fiber of 50 µm core diameter to a triple grating monochromator/spectrograph and a thermoelectrically cooled Marconi CCD camera. A frequency-doubled neodymium-doped yttrium–aluminum–garnet (Nd:YAG) laser (λ = 532 nm) and a 50X air objective lens (Nikon, Tokyo, Japan) with a numerical aperture (NA) of 0.75 were used for the current measurements. To avoid sample photodegradation, the average laser power was maintained at a low output of about 3 mW. Arrays of 150 × 150 Raman spectra, at an integration time of 500 ms per spectrum, were collected for the surface Raman mapping images of untreated and NDGA-treated GBM cells. The WITec Control software, which controls the piezoelectric stage for sample scanning, was employed for acquiring the confocal microscopic data.

### 2.3. Computational Analysis

The current statistical analysis was performed using an in-house algorithm developed in MATLAB^®^ version r2016a. Prior to implementing this algorithm, general linear background subtraction was applied to the Raman output data in the region of 377 cm^−1^ to 3500 cm^−1^. To maintain consistency between measurements of different samples and eliminate potential slight fluctuations in the laser power during the fast confocal data recording, normalization to the intensity of the laser line, whose height was derived from a Gaussian fit, was also implemented in all of the individual spectra. To further improve the accuracy of the calculations, additional linear background subtractions were also carried out for the integrated areas under the relevant Raman features. The particular frequency regions for these second background subtractions are as follows: from 980 to 1040 cm^−1^ for the phenylalanine vibrational line centered at 1004 cm^−1^; from 1190 to 1400 cm^−1^ for the amide III convoluted features centered at 1267 cm^−1^ and 1338 cm^−1^ (cancerous sample) and at 1304 cm^−1^ (non-cancerous sample); from 1400 to 1530 cm^−−1^ for the band centered at 1461 cm^−1^ (lipid, protein); from 1530 to 1750 cm^−1^ for the broadband centered at 1605 cm^−1^ (phenylalanine, reduced nicotinamide adenine dinucleotide (NADH), tryptophan, mitochondria) and the peak at 1667 cm^−1^ (amide I, β-sheet); and from 2800 to 3040 cm^−1^ for the three convoluted features centered at 2854 cm^−1^ (fatty acids, aliphatic acyl chain of endogenous lipids), 2888 cm^−1^ (lipids), and 2935 cm^−1^ (proteins), respectively. Next, for each sample, about 1000 spectra corresponding to cells were selected to calculate the ratios of different parameters related to compositional content changes. Although calculations for all the potential combinations of these parameter ratios were independently performed for each spectrum, only the ratios showing defined trends of NDGA’s influence are presented and discussed in the current work.

## 3. Results and Discussion

**Figure 1:**
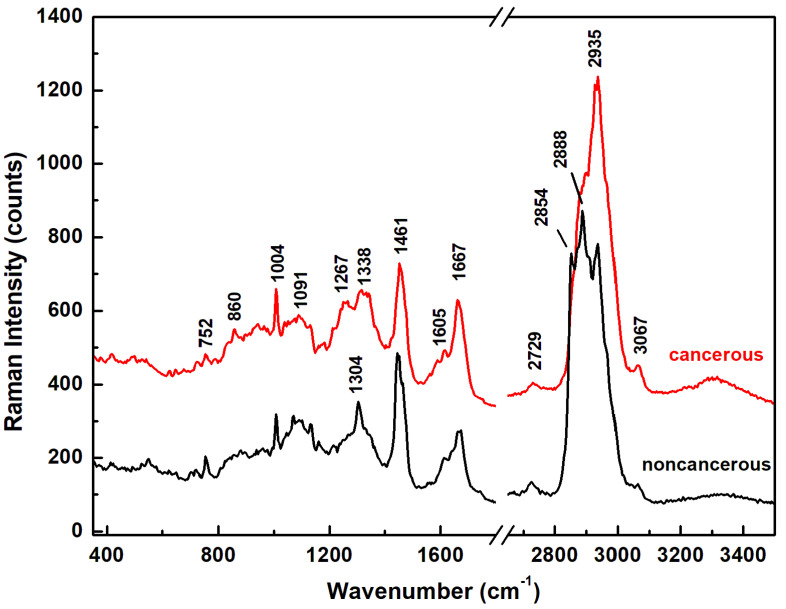
Integrated Raman spectra of normal control sample (black spectrum) and GBM cancer control sample (red spectrum).

Since pathological cell modification is accompanied by fundamental changes in cell biochemical structure, in Figure 1 we first present the Raman spectra of normal (non-cancerous) and malignant (cancerous) control samples, which were acquired from mouse brain tissue and GBM cancer cells, respectively. Each of these two representative Raman spectra was obtained by averaging tens of thousands of accumulated spectra (90,000 spectra for the current measurements). To include all the regions of interest in identifying differences in the vibrational signatures between the two control samples, a break between 1800 and 2650 cm^−1^ was applied. The spectra were also vertically translated for easier visualization and comparison. Not only do the spectra in Figure 1 show distinct differences in the intensities of some vibrational lines, which allow for accurate discrimination between normal and malignant samples, but they are also in good agreement with similar results reported in the literature [16,20]. Recognition of the main vibrational signatures that contribute to distinguishing between normal and malignant samples is the first step in facilitating a better understanding of the application of NDGA studies to GBM therapeutics.

The most significant distinction between these two Raman spectra is observed in the 2800–3000 cm^−1^ lipid-protein profile region. Certain spectroscopic features occurring in this region have already been analyzed and reported as the main indicators of carcinogenesis [16,20]. The noticeable enhancement in strength of the Raman peak at 2935 cm^−1^ observed for the GBM cancerous sample suggests a higher content of transformed protein. This observation is supported by the slight increase in the intensity of the vibrational line at 1667 cm^−1^, which is attributed to amide I (β-sheet, cholesterol esters). Another biochemical modification in the structure of proteins is associated with the small intensity decrease of the Raman feature at 1461 cm^−1^. On the contrary, a dominant lipid content is indicated by the spectrum of the normal control sample, with a sharp Raman peak at 2888 cm^−1^ and a well-defined vibration at 2854 cm^−1^ (fatty acids, aliphatic acyl chain of endogenous lipids). These two Raman vibrational lines become only a broad shoulder in the lower frequency region of the 2935 cm^−1^ peak for the GBM sample. Thus, an abnormal lipid-protein metabolism, which is known to occur in various types of cancer and to generate a post-translational modification of proteins, could be the underlying reason for these observed structural changes [44,45]. It has been suggested that this overexpression of proteins and dysregulation of signaling pathways observed in cancer metabolism originate predominantly from alteration of the cell membrane, but not exclusively [44,45]. Other corroborative indications are the obvious differences in the content of the phospholipids, protein amide III, nucleic acids, and collagen between these two control samples. For example, the sharp, strong Raman peak at 1304 cm^−1^ (lipids, phospholipids, collagen, protein amide III, and DNA) in the normal control sample splits into two broad and less intense Raman features at 1267 cm^−1^ (amide III, fatty acids, and P=O asymmetric stretch due to nucleic acids) and 1338 cm^−1^ (protein, DNA/RNA, tryptophan, and mitochondria) in the spectrum associated with the malignant sample. A broadening and intensity decrease is also seen for the vibration at 1091 cm^−1^ (cell membrane phospholipids and nucleic acids). Furthermore, with structural changes of the cells towards a malignant configuration, there is a visible enhancement in the amount of phenylalanine, which is correlated with the intensity increase of the peak at 1004 cm^−1^. Thus, besides the observed abnormal lipid-protein metabolism, overexpression of amide I, and potential transformation of the α-helix structure into a β-sheet, which were previously reported in the literature for malignant tumors [16,20,37,44,45], phenylalanine can also be considered as a biomarker of tumorigenesis. All the Raman vibrational bands observed in the spectra, along with their assignments and tentative attributions, are summarized in Table 1 below.

**Figure 2:**
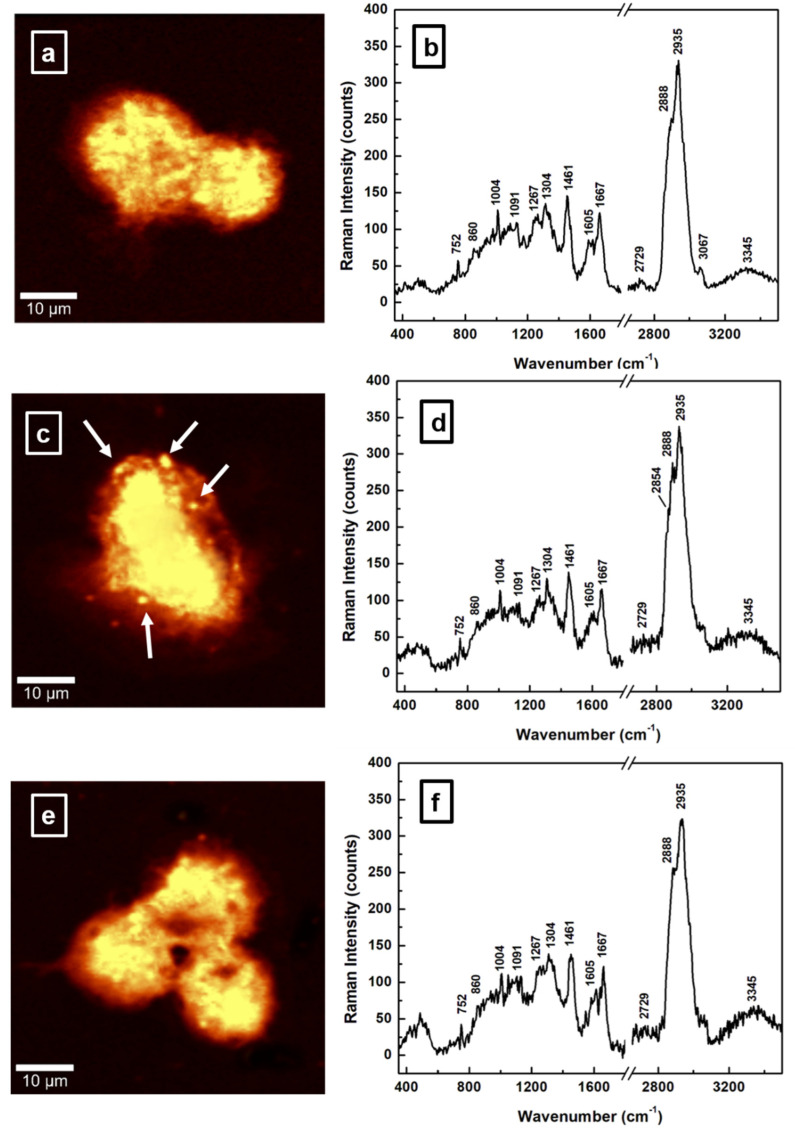
(**a**–**f**). Representative images acquired with surface confocal Raman mapping of: (**a**) untreated (control) GBM cells, (**c**) 100 µM NDGA-treated GBM sample treated for 24 h, and (**e**) 250 µM NDGA-treated GBM sample treated for 4 h. A bright yellow pseudo-color corresponds to a higher intensity. (**b**,**d**,**f**) Raman spectra associated with each image and only in vibrational regions of interest.

Concerning the main intent of this analysis to investigate the influence of NDGA in alleviating malignant brain tumors, we present, in Figure 2a,c,e, representative surface confocal Raman mapping images of an untreated (control) GBM sample and two NDGA-treated GBM samples. Amounts of 100 µM NDGA for 24 h and of 250 µM NDGA for 4 h were used for the two differently treated samples. The corresponding spectroscopic data for each image (averages over 22,500 independent spectra acquired per image) are shown in Figure 2b,d,f. The same frequency regions were considered, with a break between 1800 and 2650 cm^−1^, for easier comparison with the previously discussed Raman bio-structural signatures presented in Figure 1. Background subtraction and normalization to the intensity of the 2935 cm^−1^ vibrational line were also performed. Although direct identification of NDGA is not expected to be possible at these low compound concentrations, which are below the threshold of Raman spectroscopy detectability, NDGA administration is anticipated to induce structural modifications to the cell. Thus, by identifying such changes we can probe some of the NDGA treatment’s molecular mechanisms that are relevant to the possibility of its therapeutic use.

Comparing the integrated spectrum of the untreated sample (GBM control sample, Figure 2b) to those of the NDGA-treated samples (Figure 2d,f) and focusing on the protein to lipid content ratio, which is obtained from the I_2935_/I_2888_ intensity ratio of the corresponding Raman features, reveals a decrease in this ratio. A value of 1.32 ± 0.03 is obtained for the malignant GBM sample, of 1.20 ± 0.03 for the NDGA-treated GBM sample treated with 100 µM for 24 h, and of 1.26 ± 0.03 for the NDGA-treated GBM sample treated with 250 µM for 4 h. Since for the normal control sample, a lower value of 0.87 ± 0.03 is estimated for the I_2935_/I_2888_ ratio, this reduction of acetylated protein content implies that NDGA has a benefic effect. Further supporting evidence of NDGA’s action on the cell’s lipid-protein metabolism is the presence of the lipid droplets that are marked by white arrows in Figure 2c and that correspond spectroscopically to the very weak feature at about 2854 cm^−1^ for the sample treated with 100 µM NDGA (see Figure 2d). However, for a higher dose of NDGA over a shorter time (comparable to bolus therapy), in Figure 2f there is a lipid peak at 2888 cm^−1^ that is slightly more prominent than it is in the Raman spectrum of the GBM sample (Figure 2b). This increase in the protein to lipid ratio suggests a less benefic effect. A closer look at the image and the Raman spectrum presented in Figure 2e,f, respectively, besides revealing less evidence of lipid droplets, shows an increase in fatty acid content (see the slightly higher intensity of the 1267 cm^−1^ line), which is also a characteristic of altered metabolism in cancer [20]. Thus, a higher dosage is less recommended and toxic. It was stipulated that this de novo fatty-acid synthesis results from the Otto Warburg effect. Enhanced glucose catabolism induces an increase in pyruvate as a byproduct, which is further converted to lactate and acetyl coenzyme A (acetyl-CoA) [20]. The latter is a known component in biochemical reactions involving lipid-protein metabolism and carbohydrates. Higher NDGA dosage also induces differentiation and inhibition of self-renewal of glioma stem cells, cell membrane damage, and apoptosis [30].

Another structural modification is related to the overexpression of amide I and the transformation of an α-helix structure into a β-sheet [37]. By considering the I_1667_/I_1605_ intensity ratio of the associated Raman bands, values of 1.35 ± 0.03 for the normal control sample, 1.49 ± 0.03 for the GBM tumorigenic sample, 1.45 ± 0.03 for the NDGA-treated GBM sample treated with 100 µM for 24 h, and 1.47 ± 0.03 for the NDGA-treated GBM sample treated with 250 µM for 4 h are estimated here. These values suggest that NDGA addition does not make a significant contribution to reversing this unwanted structural transformation associated with the tumorigenic samples. On the other hand, investigation of changes in the intensity of the phenylalanine vibrational line shows a decrease from the untreated GBM sample to the NDGA-treated samples. Values of 38.2 ± 0.02 for the GBM tumorigenic sample, 37.5 ± 0.02 for the NDGA-treated sample treated with 100 µM for 24 h, 29.7 ± 0.03 for the NDGA-treated GBM sample treated with 250 µM for 4 h, and 28.5 ± 0.02 for the normal control sample were obtained.

An essential amino acid, phenylalanine, is usually taken from food and metabolically transformed into tyrosine. Unfortunately, in malignant tumors, ROS-damaged phenylalanine also occurs [46]; this statement corroborates the visible increase of the 1004 cm^−1^ vibration line in Figure 1 for the cancerous sample. The specific pathways by which such ROS-damaged amino acids incorporate into and modify the structure of proteins remain unknown. However, they could contribute to the increase of the 2935 cm^−1^ peak in Figure 1. Thus, based on the observed results, we consider that NDGA, which is a ROS scavenger and an antioxidant phenolic lignan, impairs and potentially reverses such ROS-damaged phenylalanine production.

**Figure 3:**
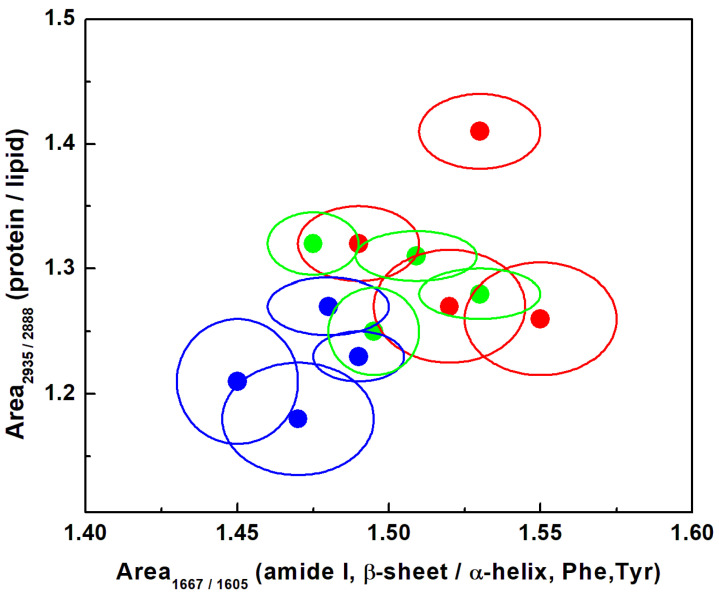
Statistical representation using 1-sigma ellipsoids of the content ratio associated with the protein to lipid contents (i.e., ratios of 2935 cm^−1^ to 2888 cm^−1^) and that of the protein, amide I β-sheet, phenylalanine, and tyrosine (i.e., ratios of 1667 cm^−1^ to 1605 cm^−1^). The solid circle defines the average over 22,500 spectra for each biomarker. A red color code was used for the malign GBM sample, blue for the NDGA-treated GBM sample treated with 100 µM for 24 h, and green for the NDGA-treated GBM sample treated with 250 µM for 4 h.

**Figure 4:**
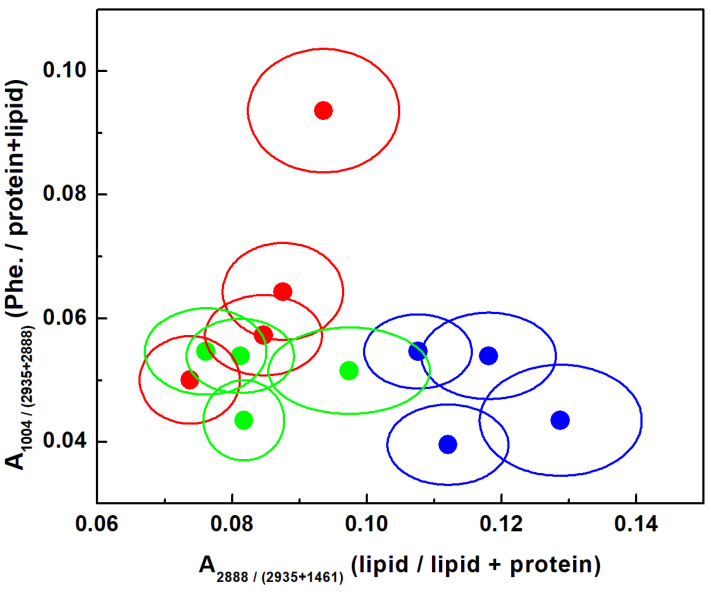
Statistical representation, using 1-sigma ellipsoids, of ratios of phenylalanine content to combined protein and lipid content (i.e., ratios of peak areas at 1004 cm^−1^ to corresponding sums obtained by adding peak areas at the 2935 cm^−1^ and 2888 cm^−1^) and corresponding ratios of lipid to overall protein content (i.e., ratios of peak areas at 2888 cm^−1^ to corresponding sums obtained by adding peak areas at 2935 cm^−1^ and 1461 cm^−1^). The solid circle defines the average over 22,500 spectra for each biomarker. A red color code was used for the malign GBM sample, blue for the NDGA-treated GBM sample treated with 100 µM for 24 h, and green for the NDGA-treated GBM sample treated with 250 µM for 4 h.

A more compact and informal visualization of the potentially beneficial influence of NDGA in reducing some of the contents that are directly associated with cell malignancy is presented in Figure 3 and Figure 4. The solid circles in the 1-sigma ellipsoid statistical representations correspond to the averages of the compound content over all the spectra. This type of representation also allows for detecting anticipated differences between different samples of the same type. The integrated areas under the peaks are considered instead of their intensities, in order to minimize the calculation errors arising from the inhomogeneity of sample roughness and to avoid the influence of polarization-sensitive effects for some of the constituents. The protein to lipid content ratio, A_2935_/A_2888_, as a function of the A_1667_/A_1605_ of protein, amide I β-sheet, phenylalanine, and tyrosine are presented in Figure 3.

Besides being in good agreement with our previous remark that administration of NDGA has a constructive effect in reducing the amount of modified protein content, inter-sample variance can also be observed. In this context, the increase in the cell fluorescence with NDGA incorporation, which is first identified through a signal to noise (S/N) ratio increase in the spectra presented in Figure 2d,f, affects the measurements by adding to the errors in discriminating between the samples. However, based on the current results, a smaller NDGA amount of 100 µM NDGA for 24 h is recommended, not only because it has potentially lower toxicity, but it enables better discrimination between the samples (comparison between 1-sigma ellipsoid blue color plots and those of red color). Another contributing factor that is worth mentioning is the expected auto-oxidation of NDGA itself and its transformation into semi-quinone or ortho-quinone forms, which exhibit vibrational frequencies in the 1600 cm^−1^ region [30,40]. Since a larger amount of NDGA incorporation would produce a larger amount of such oxidized species, less discrimination between the samples is expected and observed for A_1667_/A_1605_ (comparison of 1-sigma ellipsoids along the horizontal axis).

Statistical plots of the ratio of phenylalanine content to the combined protein and lipid content, A_1004_/A_2935+2888_, versus the ratio associated with modifications in the lipid and protein biostructures, A_2888_/A_2935+1461,_ are presented in Figure 4. Lipid-lowering and anti-lipid per-oxidation treatments have been investigated for other anti-cancer drugs, with even higher toxicological effects than NDGA. Thus, the analysis of Figure 4 can provide specific insights into NDGA’s influence on the molecular mechanism relating lipid metabolism to cancer, as well as into the inhibition of ROS-damaged phenylalanine formation. While a reduction in the ROS-damaged phenylalanine is observed from the trend along the vertical axis for both NDGA concentrations, the trend along the horizontal axis demonstrates that NDGA administration at a higher dose induces less lipid development. This latter remark supports our previous observation of more lipid droplet development for cell treatment with 100 µM NDGA for 24 h (see Figure 2c), with less at a higher dose. The likely structural cell damage driven by NDGA’s cytotoxicity at higher dosage should also be taken into account.

**Figure 5:**
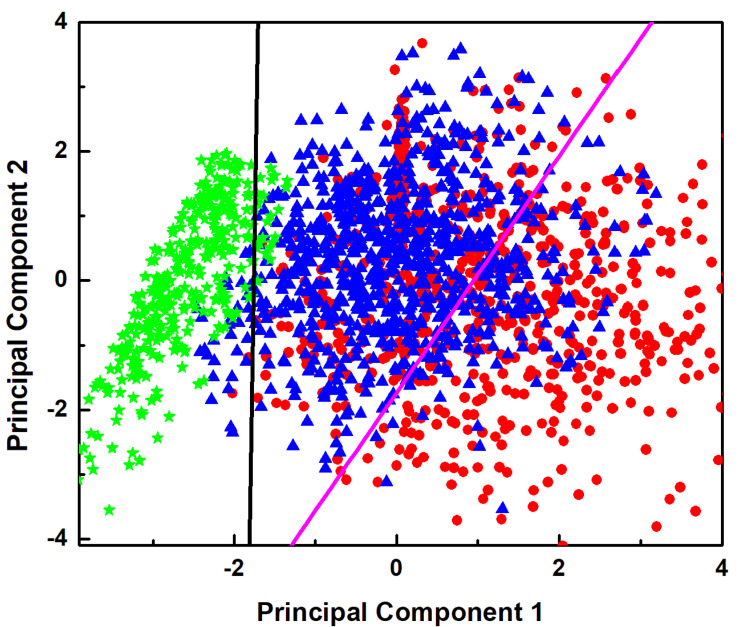
Principal component analysis (PCA) showing a clear separation between the clusters of data points of the samples. For consistency, the same color codes of red, blue, and green were used for the malign GBM sample, the NDGA-treated GBM sample treated with 100 µM for 24 h, and the NDGA-treated GBM sample treated with 250 µM for 4 h, respectively.

To further evaluate the experiments’ capability of differentiating between the untreated and NDGA-treated cells we performed principal component analysis (PCA). All of the observed Raman vibrational lines and their ratios were considered as variables in performing the PCA. An advantage of employing PCA is that it avoids introducing a bias related to a priori knowledge since PCA does not take into account the known classification of the spectra. A reduction in the dimensionality of the system was implemented to improve the visualization of the results, which are presented in Figure 5. The first two principal components contain about 78% of the total variance of all the samples. In addition, for consistency with the data of previous figures, an identical color-code was used, with red representing GBM samples, blue denoting the NDGA-treated GBM samples treated with 100 µM for 24 h, and green for the NDGA-treated GBM sample treated with 250 µM for 4 h. The main observation derived from this figure is that the clusters of data points of the three types of samples are separated, with some overlapping between those of the untreated GBM and NDGA-treated-with-100 µM samples. Linear discriminant analysis (LDA) was used for sample classification.

Since a dosage higher than 100 µM NDGA is reported as toxic [30], besides not recommending it, we performed additional assessment using machine-learning techniques to build a fully automated framework that makes decisions directly from Raman spectra given in input. To discriminate better between the cell samples, we employed all the ratios as variables of the input training for various statistical learning algorithms, such as Support Vector Machines (SVM), k-Nearest Neighbor (kNN), Decision Tree Learning (DTL), and Naïve Bayes Classifiers (NBC), using five-fold cross-validation. Classification accuracy of about 80% resulted from all these statistical learning algorithms. The confusion matrix for the Linear Support Vector Machine (LSVM) is presented in Table 2 below.

This table reveals that the algorithm’s capability to classify a single spectrum (true positive) is 68.2% for untreated GBM samples, 81.2% for samples treated with 100 μM NDGA, and 95.2% for samples treated with 250 μM NDGA. It also shows a 31.8% misclassification of the untreated GBM samples as those treated with 100 μM NDGA. Among the samples treated with 100 μM NDGA, 16.8% are misclassified as untreated GBM and 2.0% as treated with 250 μM NDGA. The 250 μM NDGA-treated samples are separated much more clearly, with no such misclassification as untreated; only 4.8% are misclassified as treated with 100 μM NDGA. These results corroborate those shown in Figure 5. They also confirm that more than a single spectrum is necessary to have accurate discrimination between the samples.

One of the advantages of Raman microscopy is the simultaneous recording of multiple spectra from the same sample, which, from the perspective of statistics, can be interpreted as independent sampling (each spectrum is recorded at a slightly different position). Assuming that *p* is the true positive rate and (1 − *p*) the false-negative rate, the probability of misclassification is provided by [47]:

(1) $$Q\left(N\right)=1-\overset{k<\frac{N}{2}}{\underset{k=0}{\sum }}\left(\begin{array}{c} N\\ k \end{array}\right){\left(p\right)}^{N-k}{\left(1-p\right)}^{k}$$

where *N* is the number of independent spectra measured from the same sample and *k* is the number of spectra not associated with a category (0 < *k* < *N*/2).

**Figure 6:**
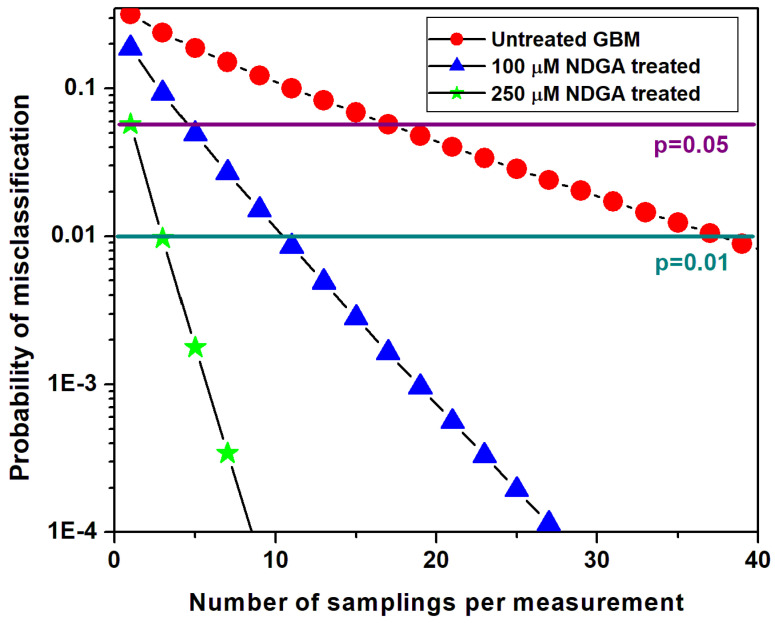
Probability of misclassification versus the number of randomly chosen spectra employed in the classification. The horizontal violet and dark cyan lines are for easier visualization of the sets of measured spectra sufficient to classify the samples with error probabilities of *p* = 0.05 and *p* = 0.01, respectively.

These probabilities for sample misclassification after *N* Raman recordings are provided in Figure 6. For easier visualization of the set of measurements sufficient to accurately classify the samples, we include in this figure two horizontal lines representing error probabilities of *p* = 0.05 and of *p* = 0.01. About 21 spectra will be enough to classify the samples with an accuracy of 95% and 41 spectra are needed for an accuracy of 99%. These numbers are very small compared to the capability of confocal Raman microscopy to provide on the order of 10,000 independent spectra per sample. Not only is the classification accuracy of this method extremely good, but, more importantly, the Raman technique has the potential for future in vivo applications.

## 4. Conclusions

The purpose of this study has been to demonstrate the capability of Raman microscopy for detecting structural differences in GBM cells before and after treatment with NDGA, which contributes to understanding the compound’s potential in alleviating brain tumors. Besides experimental Raman analysis, the computational approach employed helps to discriminate between malignant and benign brain tumor biospecimens and to identify minute structural changes in GBM’s bio-signatures upon NDGA administration. A prior examination of the main vibrational signatures that contribute to distinguishing between normal and malignant samples was considered necessary for a comprehensive understanding of NDGA’s contribution to GBM therapeutics. The current results show benefic effects of NDGA in reducing the amounts of altered protein content and ROS-damaged phenylalanine. It is worth emphasizing here that phenylalanine, in addition to other known cancer biomarkers, can be used for sample classification and for assessing NDGA’s efficacy. Providing repetitive, smaller dosages of NDGA over a longer time, similar to a quasi-metronomic type of therapy, has been demonstrated to be a better therapeutic approach.

Another important observation relates to the abnormal lipid-protein metabolism associated with various types of cancer and the formation of lipid droplets. Again, treatment with a lower NDGA dosage is recommended, as very high doses of NDGA, similar to a quasi-chemotherapy approach, induce membrane and other structural cell damage. However, the known detrimental cytotoxicity of NDGA in high doses for prolonged periods of time might have some beneficial chemotherapeutic effects if employed as a bolus therapy. Further work needs to be done to investigate this assumption, as well as to analyze the efficiency of other possible NDGA chemical derivatives for such therapeutic applications. In the future, we plan to perform a double-blind analysis on such samples, by independently using our algorithm and Raman method and complementary standard bioanalysis, to investigate how the results derived from both approaches compare. To get a step closer to potential in vivo implementation of our spectroscopic method and our statistical algorithm in assessing GBM as a disease, fast acquisition of random Raman spectra numbering on the order of a hundred are planned for evaluation of the number of instances in which the NDGA-treated and untreated samples would be found statistically significantly different (at the *p* = 0.05 level).

This work is in itself of substantial value since it creates the needed foundation and awareness of NDGA’s beneficial and detrimental mechanisms of action with a view towards brain cancer therapy. By correlating our results with future in vivo studies of NDGA’s bio-activity, we anticipate that continuous and accelerated progress for new drug development can be accomplished.

## Figures and Tables

**Table 1:** Raman vibrational bands and their assignments with tentative attributions.

Raman Wavenumber cm^−1^	Assignment ^a,b^	Tentative Attribution ^a,b^
752	CH_2_ rocking, symmetric breathing	Tryptophan, cytochrome c, mitochondria ^a^
		Nucleic acids, tryptophan ^b^
860	CC stretch	Tyrosine, proline, glycogen ^b^
1004	Symmetric CC aromatic ring breathing	Phenylalanine, collagen IV, I ^a^ Phenylalanine ^b^
1091	CC skeletal stretch, PO_2_ symmetric stretch	Protein, phospholipids, glycogen, collagen IV, I ^a^ Phospholipids, nucleic acids ^b^
1267	Amide III, =C–H bend, P=O asymmetric stretch	Homo polypeptide ^a^, Fatty acids ^b^
1304	Amide III, N–H bend, α-helix, C–N stretch, and CH_3_ bend, C–H^2^ twist	Bending and stretching coupled in-phase, collagen IV, I ^a^ Lipids, phospholipids, collagen, protein, DNA ^b^
1338	CH_2_ deformation	Protein, A and G of DNA/RNA ^a^ Tryptophan ^b^
1461	CH_2_ or CH_3_ out-of-phase deformation, CN bend	Lipid, protein ^a^ Protein ^b^
1605	Amide I α-helix, CO stretch, C=C bend	Protein, phenylalanine, tyrosine ^a^ Unsaturated fatty acids, triglycerides ^b^
1667	Amide I β-sheet, C=O stretch	Unordered or random structure, collagen IV, I ^a^ Proteins, cholesterol esters ^b^
2729	CH_3_ in-phase deformation overtone	
2854	CH_2_ symmetric stretch	Fatty acids, triglycerides ^a,b^
2888	CH_2_ asymmetric stretch	Lipids ^a,b^
2935	CH_3_ symmetric stretch	Proteins ^a,b^
3067	CH_3_–(C=O), C–H aromatic	Nucleic acids, proteins ^b^

^a,b^ Refs. [16,20], and all references therein.

**Table 2:** Classification accuracy using 5-fold cross validation LSVM.

**True**			**Predicted**	
		**Untreated GBM**	**100 µM NDGA-Treated**	**250 µM NDGA-Treated**
	**Untreated GBM**	68.2%	31.8%	0%
	**100 µM NDGA-treated**	16.8%	81.2%	2.0%
	**250 µM NDGA-treated**	0 %	4.8%	95.2%
